# Pregnancy follow-up in a patient with mechanical valve: possible in sub-Saharan Africa?

**Published:** 2009-03-06

**Authors:** Jacques Cabral Tantchou Tchoumi, Jean Claude Ambassa, Gianfranco Butera

**Affiliations:** 1 Shisong Cardiac Centre, Kumbo, Cameroon; 2 Istituto Policlinico San Donato, Milan, Italy

**Keywords:** Pregnancy, mechanical prosthesis

## Abstract

**Background:**

In Africa in general and in Cameroon in particular, post rheumatic cardiopathies are a health care problem, one of the causes of infertility in the women population and a major cause of death among children and adults. Management of a pregnant woman with mechanical heart valve is a complex issue for all health care providers involved in the care of such patients.

**Patient and case report:**

Miss A is 26-years old and consulted for cardiac assessment; referred from Bamenda (North-West province of Cameroon) for better management of a cardiac problem including arrhythmia and a history of recurrent tonsillitis. The cardiac echo-dopplerography showed severe post-rheumatic mitral valve regurgitation with pulmonary hypertension and a dysfunctional left ventricle. The patient was later evacuated in a surgical centre in Milan San Donato (Italy) where a St. Jude mechanical heart valve N.27 was implanted. Two years after surgery, during a follow-up visit, the patient brought a pelvic ultrasound showing a single live intrauterine foetus, gestational age estimated at 7 weeks.

**Conclusion:**

Management of mechanical valve in a pregnancy context, resulting in a favourable outcome (no thromboembolic events and the delivery of a healthy baby) is possible in sub-Saharan Africa. Close observation, adherence to existing clinical guidelines, patient cooperation and an appropriate technical infrastructure are critical factors to consider.

## Background

In Africa in general and in Cameroon in particular, post rheumatic cardiopathies are a health care problem and one of the causes of infertility in the women population. It is also a major cause of death among children and adults in developing countries and sub-Saharan African specifically [1]. Management of a pregnant woman with mechanical heart valve is a complex issue for all health care providers involved in the care of such patients [2]. Clinicians caring for pregnant women with prosthetic valves are faced with a dilemma when trying to provide optimal treatment. Inadequate anticoagulant therapy can result in thrombosis of the mechanical prosthetic valve while, on the other hand, anticoagulant therapy is associated with foetal and maternal bleeding and teratogenic effects [3]. The present report describes a case of pregnancy in a patient with a mitral mechanical valve.

## Patient and case report

Miss A is 26-years old and consulted for cardiac assessment; referred from Bamenda (North-West province of Cameroon) for better management of a cardiac problem including arrhythmia and a history of recurrent tonsillitis. On initial assessment, the patient showed dyspnoea II-III NYHA (New York Heart association classification), swelling of the lower limbs and palpitations. On physical examination, she was afebrile, no pallor, S_1_S_2_ irregularly irregular with 3/6 holosystolic murmur at the mitral valve area irradiating to the left anterior axillary line, good air entry bilaterally, distended abdomen with shifting dullness, not tender hepatomegaly, lower limbs with mild pitting oedema. The electrocardiogram showed atrial fibrillation with ventricular response varying from 90 to 150 beats per minute and a left ventricular hypertrophy. The cardiac echo-dopplerography showed severe post-rheumatic mitral valve regurgitation with pulmonary hypertension and a dysfunctional left ventricle. The patient received the conventional treatment of heart failure and there was a strong indication for mitral valve replacement.

The patient was later evacuated in a surgical centre in Milan San Donato (Italy). After clinico-medical discussion and patient’s consent, a St. Jude mechanical heart valve N.27 was implanted. Two years after surgery, during a follow-up visit, the patient brought a pelvic ultrasound showing a single live intrauterine gestation, gestational age estimated at 7 weeks. Acecoumadin was immediately discontinued for low molecular weight heparin (LMWH) enoxaparin, 0,4 ml twice daily subcutaneously, prescribed for 6 weeks, from gestational week 8 to 14. LMWH was given blindly because of the impossibility to control Factor anti-Xa. The next appointment was given for the end of the 14^th^ week of pregnancy. For some reasons that we couldn’t understand, the patient came back only at gestational week 17. LMWH was replaced by acecoumadin and we maintained the International Normalised Ratio (INR) between 2.5 and 3. A cardiac echography was done at the beginning and at the end of the therapy with LMWH and every month thereafter, as well as a pelvic ultrasound. Cardiac echocardiographies were without major changes. At the beginning of the 36^th^ week of gestation, the patient was admitted for an elective caesarean section (CS). Acecoumadin was discontinued and replaced by enoxaparin two days before admission for elective caesarean procedure. A baby boy was delivered; weight 2800 grams, height 30 centimetres, Apgar 7 and no apparent foetopathy on clinical examination.

## Discussion

This is the very first case of pregnancy in a patient with a mechanical valve since the opening of the Cardiac Centre in St Elizabeth Catholic General Hospital of Shisong (North Eastern province of Cameroon). In this case, we reported that to prevent thromboembolic events and foetopathies, acecoumadine was discontinued at week 7 of gestation because of the late presentation of the patient, followed by LMWH for 9 weeks. The revised American Heart Association and American College of Cardiology (AHA/ACC) 1998 guidelines for management of patients with valvular heart disease stipulates that acecoumadine has to be changed with continuous unfractioned heparin or dose-adjusted unfractioned heparin, or dose-adjusted LMWH from the 6th till the 12^th^ week of gestation. The result we got is similar to the one of Ueno M. et al. [4] who opted to undergo an alternative approach involving the use of antiplatelet agents (dipyridamole, ticlopidine and aspirin) in place of warfarin potassium. Antiplatelet agents were administered while regularly monitoring the platelet aggregability along with coagulation and fibrinolytic activity. At week 36 of gestation, antiplatelet agents were discontinued and followed by continuous heparin infusion. Our patient decided later, on her own, to continue with LMWH till the 17^th^ week of gestation and acecoumadin was then reinstated from the 18th week till 2 days before the elective CS (at the end of the 36^th^ week of gestation). It is important to mention that patient illiteracy is sometimes a major issue we face in the follow-up of difficult cases in Africa; more than half of our patients didn’t finish secondary school. No clear reason was given for the missed appointment. However, the patient showed no side-effects or complications of the prescribed therapy. Ben Ismail et al [5] reported the difficulties of achieving accurate anticoagulation in Tunisia.

Two out of five women given heparin had thromboembolic episodes and three had spontaneous abortions. Oral anticoagulants were given during 53 pregnancies: there were eight spontaneous abortions but no embryopathies. Ben Ismail et al did not think that the use of heparin in the first trimester was justified. We didn’t observe any foetopathy despite the fact that acecoumadin was discontinued at the end of the 7th week of gestation instead of the 6th week as cited in the latest AHA/ACC guidelines for follow-up of pregnancy in patients with mechanical valve. Not very sure of the adequate dosage of the LMWH and not being able to control the factor Xa made the management of this case really challenging. Fortunately, no abortion or bleeding was observed from the beginning till the end of the pregnancy with the administered dosage. In the study of 47 pregnancies in 37 patients with prosthetic valves reported from India [6], oral anticoagulants were continued throughout pregnancy and replaced by heparin before labour. Forty infants were born at full term and three were premature; there were two spontaneous abortions, one stillbirth, and one ectopic pregnancy and overall foetal mortality of 8-5%. In addition, no embryopathies were observed.

It appeared that, in India or in Cameroon the medical staffs faces numerous logistic problems and have to find a way out, using the available minimum to perform great things. Sareli *et al.* [7] reported no maternal thromboembolic complications or deaths among 49 South African patients with 60 mechanical valves who were treated with warfarin. In their study, because of late presentation, no patients had received heparin in the first trimester: at week 23 of gestation, heparin was replaced by warfarin and a stillbirth was occurred at 36^th^ week; the remaining 18 patients went into labour prematurely while they were still under warfarin: in total there were six stillbirths. In addition, two warfarin related embryopathies (4%) were observed with nasal hypoplasia in both infants and stippled epiphyses in one. Sareli *et al.* concluded that with the new generation of mechanical valves (Medtronic Hall and St. Jude Medical) the maternal risk is low and, although foetal wastage is high, the risk of malformation in live born infants remains low; this statement can probably explain the good outcome of our case. Vuval KM *et al.* [8] reported among 30 patients receiving uninterrupted low-dose oral warfarin plus aspirin throughout pregnancy, three normal deliveries, two premature births, one low birth weight, seven spontaneous abortions, and 17 therapeutic abortions.

By contrast, among 8 patients who discontinued anticoagulation despite medical advice, seven had normal-term deliveries without thromboembolic complications, and spontaneous abortion occurred in one patient. Of the 5 women taking low molecular-weight heparin regimen, three had normal deliveries; one had a premature birth, and one experienced an abortion. Two patients who were taking warfarin, subsequently replaced by heparin in the last two weeks of the first trimester had term deliveries. It appears that in almost all cases there were different approaches in the care of pregnant women with prosthetic valve and guidelines for the medical staff were not quite clear [9]. New information is given in the ACC/AHA 2006 guidelines for the management of patients with valvular heart disease [10].

## Conclusion

Management of mechanical valve in a pregnancy context, resulting in a favourable outcome (no thromboembolic events and the delivery of a healthy baby) is possible in sub-Saharan Africa. Close observation, adherence to existing clinical guidelines, patient cooperation and an appropriate technical infrastructure are critical factors to consider.

## References

[1] Abdel Moula AM, Sheriff AA (1998). Prevalence of rheumatic heart disease among school children in Alexandria, Egypt: a prospective epidemiological study. J Egypt Public Health Assoc.

[2] Naqvi TZ, Foster E (2002). Anticoagulation during pregnancy. Curr Womens Health Rep.

[3] Lavoie JP, Leduc L, Mercier LA (2004). Embolic myocardial infarction in a pregnant woman with a mechanical heart valve on low molecular weight heparin. Can J Cardiol.

[4] Ueno M, Masuda H (2001). Antiplatelet therapy for a pregnant woman with a mechanical aortic valve: report of a case. Surg Today.

[5] Ben Ismail M, Fekih M, Taktak M (1979). Prostheses valvulaires cardiaques et grossesse. Arch Mal Coeur.

[6] Cecilia M, Oakley (1995). Anticoagulants in pregnancy. Br Heart J.

[7] Sareli P, England MJ, Berk MR (1989). Maternal and fetal sequelae of anticoagulation during pregnancy in patients with mechanical heart valve prostheses. JACC.

[8] Vuval KM, Ozatik MA (2003). Pregnancy after mechanical mitral valve replacement. J Heart Valve Dis..

[9] Van Kuilenburg JT, Verheutg FW, Van Digk AP (2007). Prosthetic heart valve thrombosis, anticoagulation and pregnancy: a case report and review of the literature. Neth heart J.

[10] Writing Committee to Revise the 1998 Guidelines for the Management of Patients With Valvular Heart Disease ACC/AHA 2006 (2006). Guidelines for the Management of Patients With Valvular Heart Disease A Report of the American College of Cardiology/American Heart Association Task Force on Practice Guidelines (Writing Committee to Revise the 1998 Guidelines for the Management of Patients With Valvular Heart Disease). Journal of the American College of Cardiology.

